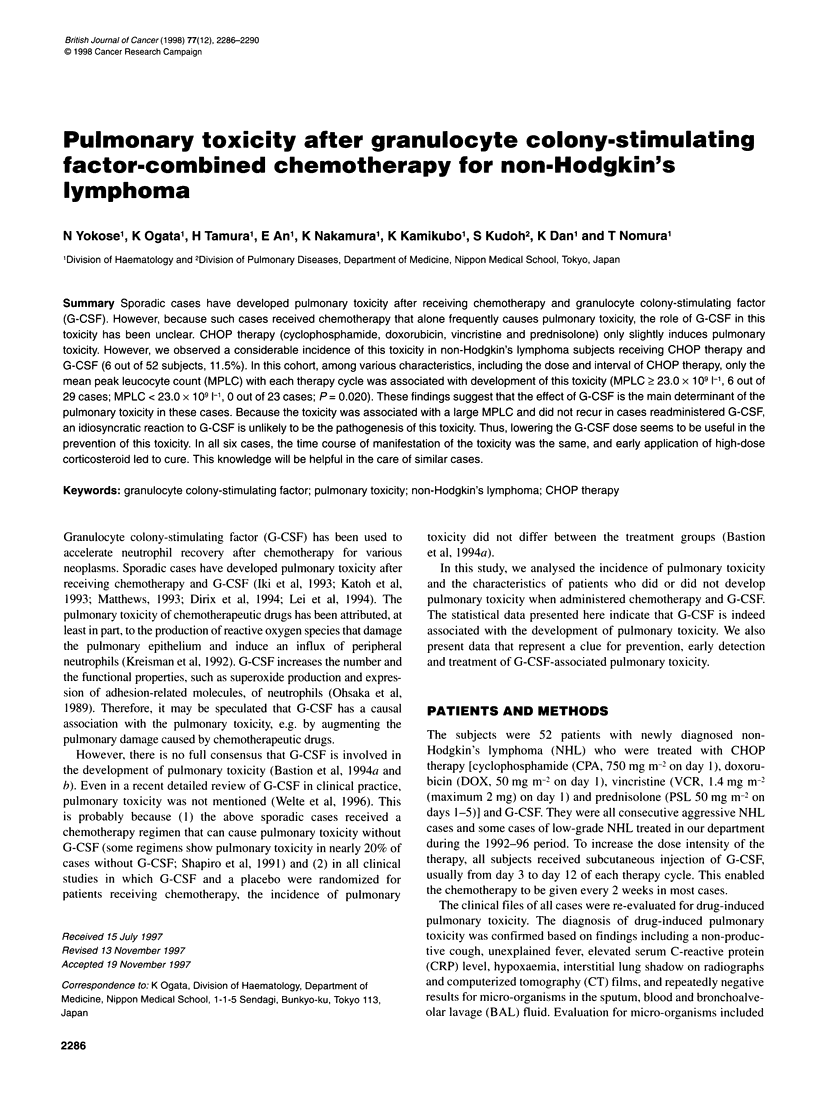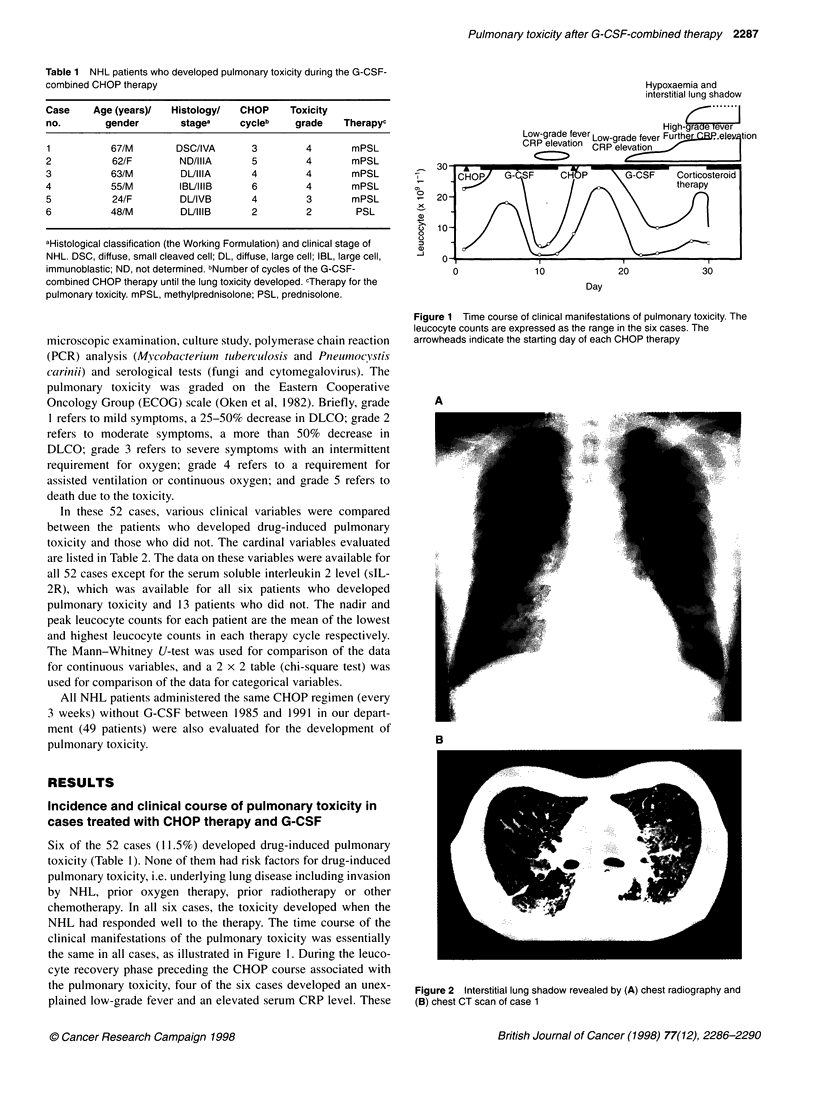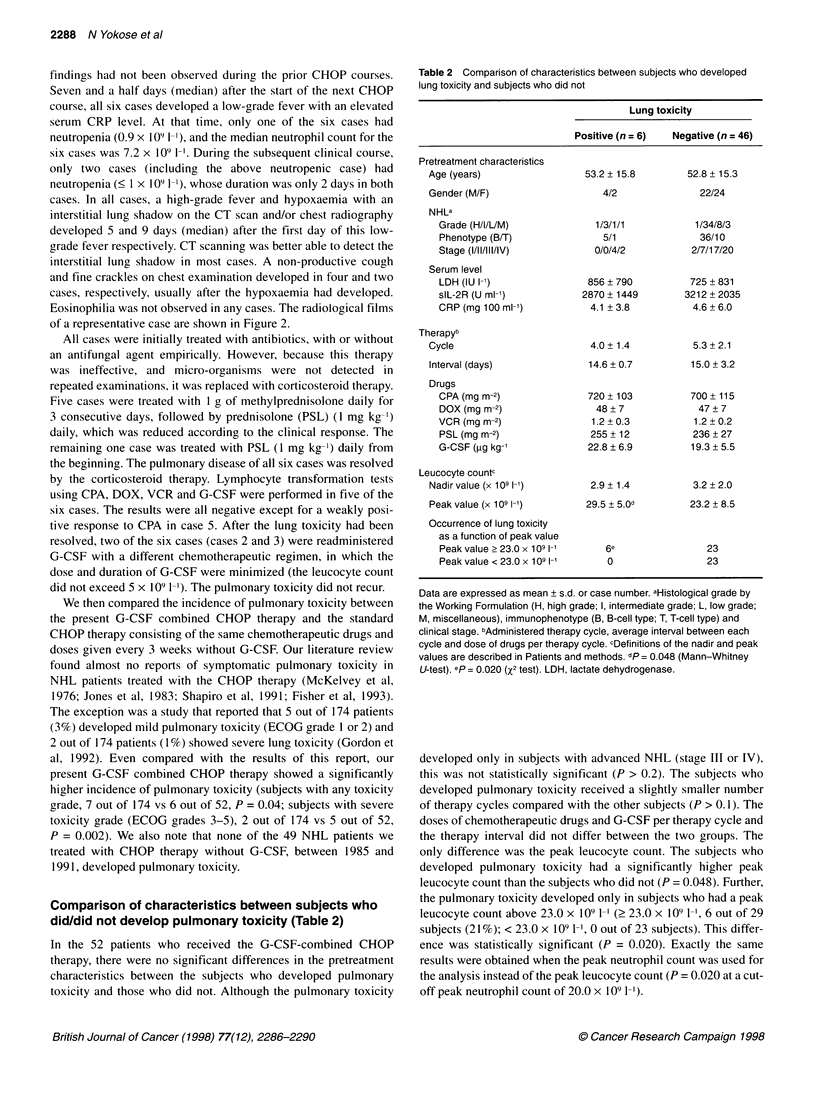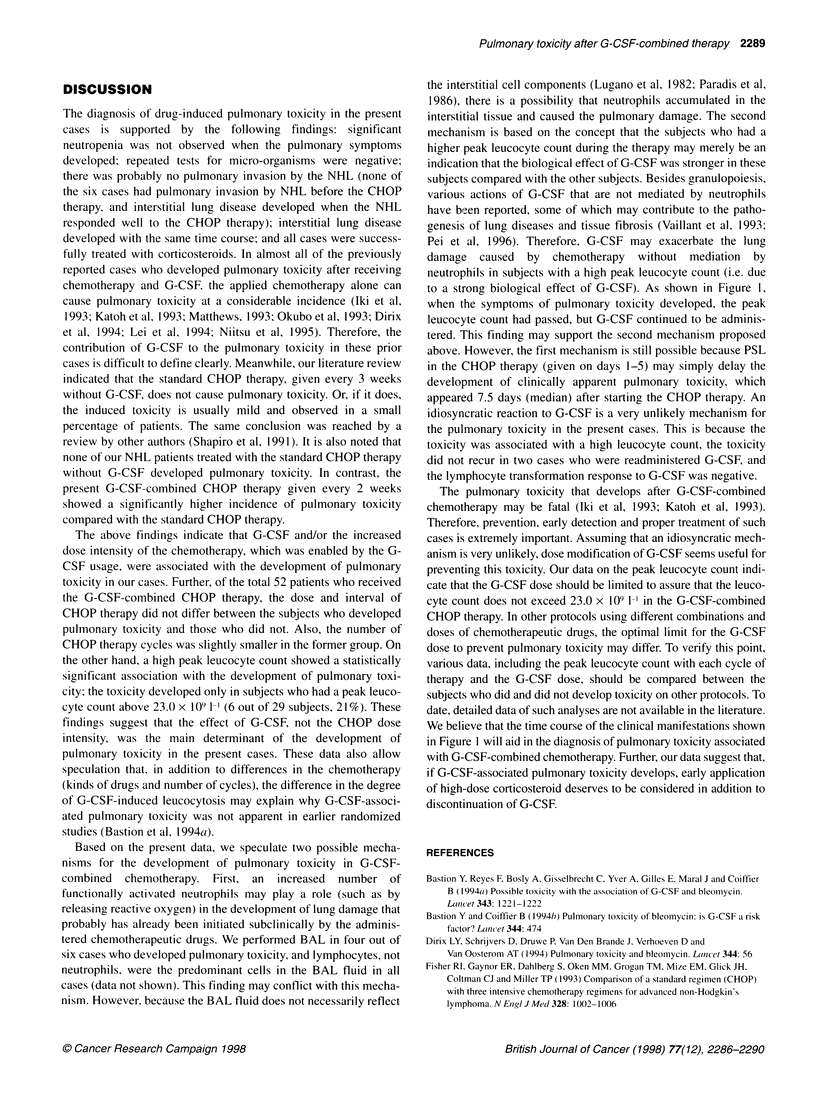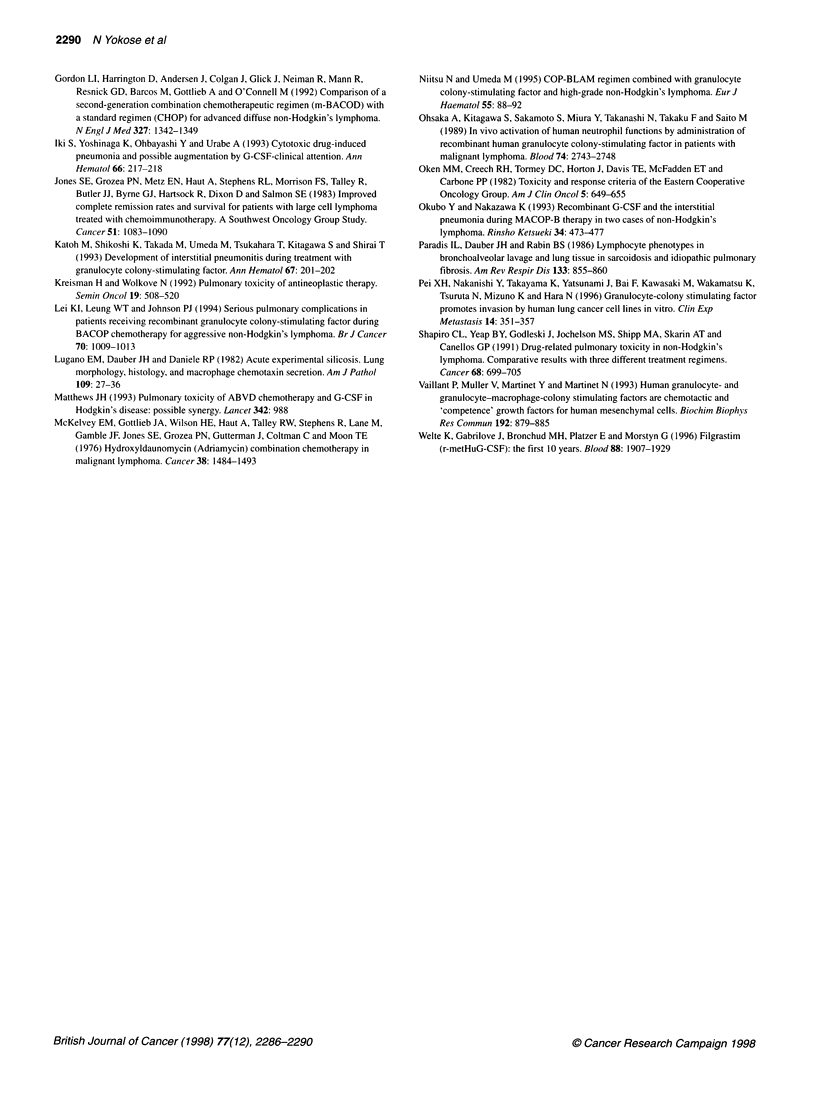# Pulmonary toxicity after granulocyte colony-stimulating factor-combined chemotherapy for non-Hodgkin's lymphoma.

**DOI:** 10.1038/bjc.1998.380

**Published:** 1998-06

**Authors:** N. Yokose, K. Ogata, H. Tamura, E. An, K. Nakamura, K. Kamikubo, S. Kudoh, K. Dan, T. Nomura

**Affiliations:** Department of Medicine, Nippon Medical School, Tokyo, Japan.

## Abstract

**Images:**


British Journal of Cancer (1998) 77(12), 2286–2290
© 1998 Cancer Research Campaign

### Pulmonary toxicity after granulocyte colony-stimulating factor-combined chemotherapy for non-Hodgkin's lymphoma

N Yokose[1], K Ogata[1], H Tamura[1], E An[1], K Nakamura[1], K Kamikubo[1], S Kudoh[2], K Dan[1] and T Nomura[1]

[1]Division of Haematology and [2]Division of Pulmonary Diseases, Department of Medicine, Nippon Medical School, Tokyo, Japan

**Summary** Sporadic cases have developed pulmonary toxicity after receiving chemotherapy and granulocyte colony-stimulating factor (G-CSF). However, because such cases received chemotherapy that alone frequently causes pulmonary toxicity, the role of G-CSF in this toxicity has been unclear. CHOP therapy (cyclophosphamide, doxorubicin, vincristine and prednisolone) only slightly induces pulmonary toxicity. However, we observed a considerable incidence of this toxicity in non-Hodgkin's lymphoma subjects receiving CHOP therapy and G-CSF (6 out of 52 subjects, 11.5%). In this cohort, among various characteristics, including the dose and interval of CHOP therapy, only the mean peak leucocyte count (MPLC) with each therapy cycle was associated with development of this toxicity (MPLC $\geq 23.0 \times 10^9$ $l^{-1}$, 6 out of 29 cases; MPLC $< 23.0 \times 10^9$ $l^{-1}$, 0 out of 23 cases; $P = 0.020$). These findings suggest that the effect of G-CSF is the main determinant of the pulmonary toxicity in these cases. Because the toxicity was associated with a large MPLC and did not recur in cases readministered G-CSF, an idiosyncratic reaction to G-CSF is unlikely to be the pathogenesis of this toxicity. Thus, lowering the G-CSF dose seems to be useful in the prevention of this toxicity. In all six cases, the time course of manifestation of the toxicity was the same, and early application of high-dose corticosteroid led to cure. This knowledge will be helpful in the care of similar cases.

**Keywords:** granulocyte colony-stimulating factor; pulmonary toxicity; non-Hodgkin's lymphoma; CHOP therapy

Granulocyte colony-stimulating factor (G-CSF) has been used to accelerate neutrophil recovery after chemotherapy for various neoplasms. Sporadic cases have developed pulmonary toxicity after receiving chemotherapy and G-CSF (Iki et al, 1993; Katoh et al, 1993; Matthews, 1993; Dirix et al, 1994; Lei et al, 1994). The pulmonary toxicity of chemotherapeutic drugs has been attributed, at least in part, to the production of reactive oxygen species that damage the pulmonary epithelium and induce an influx of peripheral neutrophils (Kreisman et al, 1992). G-CSF increases the number and the functional properties, such as superoxide production and expression of adhesion-related molecules, of neutrophils (Ohsaka et al, 1989). Therefore, it may be speculated that G-CSF has a causal association with the pulmonary toxicity, e.g. by augmenting the pulmonary damage caused by chemotherapeutic drugs.

However, there is no full consensus that G-CSF is involved in the development of pulmonary toxicity (Bastion et al, 1994*a* and *b*). Even in a recent detailed review of G-CSF in clinical practice, pulmonary toxicity was not mentioned (Welte et al, 1996). This is probably because (1) the above sporadic cases received a chemotherapy regimen that can cause pulmonary toxicity without G-CSF (some regimens show pulmonary toxicity in nearly 20% of cases without G-CSF; Shapiro et al, 1991) and (2) in all clinical studies in which G-CSF and a placebo were randomized for patients receiving chemotherapy, the incidence of pulmonary toxicity did not differ between the treatment groups (Bastion et al, 1994*a*).

In this study, we analysed the incidence of pulmonary toxicity and the characteristics of patients who did or did not develop pulmonary toxicity when administered chemotherapy and G-CSF. The statistical data presented here indicate that G-CSF is indeed associated with the development of pulmonary toxicity. We also present data that represent a clue for prevention, early detection and treatment of G-CSF-associated pulmonary toxicity.

*Received 15 July 1997*
*Revised 13 November 1997*
*Accepted 19 November 1997*

*Correspondence to:* K Ogata, Division of Haematology, Department of Medicine, Nippon Medical School, 1-1-5 Sendagi, Bunkyo-ku, Tokyo 113, Japan

### PATIENTS AND METHODS

The subjects were 52 patients with newly diagnosed non-Hodgkin's lymphoma (NHL) who were treated with CHOP therapy [cyclophosphamide (CPA, 750 mg $m^{-2}$ on day 1), doxorubicin (DOX, 50 mg $m^{-2}$ on day 1), vincristine (VCR, 1.4 mg $m^{-2}$ (maximum 2 mg) on day 1) and prednisolone (PSL 50 mg $m^{-2}$ on days 1–5)] and G-CSF. They were all consecutive aggressive NHL cases and some cases of low-grade NHL treated in our department during the 1992–96 period. To increase the dose intensity of the therapy, all subjects received subcutaneous injection of G-CSF, usually from day 3 to day 12 of each therapy cycle. This enabled the chemotherapy to be given every 2 weeks in most cases.

The clinical files of all cases were re-evaluated for drug-induced pulmonary toxicity. The diagnosis of drug-induced pulmonary toxicity was confirmed based on findings including a non-productive cough, unexplained fever, elevated serum C-reactive protein (CRP) level, hypoxaemia, interstitial lung shadow on radiographs and computerized tomography (CT) films, and repeatedly negative results for micro-organisms in the sputum, blood and bronchoalveolar lavage (BAL) fluid. Evaluation for micro-organisms included

**Table 1** NHL patients who developed pulmonary toxicity during the G-CSF-combined CHOP therapy

| Case no. | Age (years)/ gender | Histology/ stage[a] | CHOP cycle[b] | Toxicity grade | Therapy[c] |
|---|---|---|---|---|---|
| 1 | 67/M | DSC/IVA | 3 | 4 | mPSL |
| 2 | 62/F | ND/IIIA | 5 | 4 | mPSL |
| 3 | 63/M | DL/IIIA | 4 | 4 | mPSL |
| 4 | 55/M | IBL/IIIB | 6 | 4 | mPSL |
| 5 | 24/F | DL/IVB | 4 | 3 | mPSL |
| 6 | 48/M | DL/IIIB | 2 | 2 | PSL |

[a]Histological classification (the Working Formulation) and clinical stage of NHL. DSC, diffuse, small cleaved cell; DL, diffuse, large cell; IBL, large cell, immunoblastic; ND, not determined. [b]Number of cycles of the G-CSF-combined CHOP therapy until the lung toxicity developed. [c]Therapy for the pulmonary toxicity. mPSL, methylprednisolone; PSL, prednisolone.

microscopic examination, culture study, polymerase chain reaction (PCR) analysis (*Mycobacterium tuberculosis* and *Pneumocystis carinii*) and serological tests (fungi and cytomegalovirus). The pulmonary toxicity was graded on the Eastern Cooperative Oncology Group (ECOG) scale (Oken et al, 1982). Briefly, grade 1 refers to mild symptoms, a 25–50% decrease in DLCO; grade 2 refers to moderate symptoms, a more than 50% decrease in DLCO; grade 3 refers to severe symptoms with an intermittent requirement for oxygen; grade 4 refers to a requirement for assisted ventilation or continuous oxygen; and grade 5 refers to death due to the toxicity.

In these 52 cases, various clinical variables were compared between the patients who developed drug-induced pulmonary toxicity and those who did not. The cardinal variables evaluated are listed in Table 2. The data on these variables were available for all 52 cases except for the serum soluble interleukin 2 level (sIL-2R), which was available for all six patients who developed pulmonary toxicity and 13 patients who did not. The nadir and peak leucocyte counts for each patient are the mean of the lowest and highest leucocyte counts in each therapy cycle respectively. The Mann–Whitney *U*-test was used for comparison of the data for continuous variables, and a 2 × 2 table (chi-square test) was used for comparison of the data for categorical variables.

All NHL patients administered the same CHOP regimen (every 3 weeks) without G-CSF between 1985 and 1991 in our department (49 patients) were also evaluated for the development of pulmonary toxicity.

### RESULTS

### Incidence and clinical course of pulmonary toxicity in cases treated with CHOP therapy and G-CSF

Six of the 52 cases (11.5%) developed drug-induced pulmonary toxicity (Table 1). None of them had risk factors for drug-induced pulmonary toxicity, i.e. underlying lung disease including invasion by NHL, prior oxygen therapy, prior radiotherapy or other chemotherapy. In all six cases, the toxicity developed when the NHL had responded well to the therapy. The time course of the clinical manifestations of the pulmonary toxicity was essentially the same in all cases, as illustrated in Figure 1. During the leucocyte recovery phase preceding the CHOP course associated with the pulmonary toxicity, four of the six cases developed an unexplained low-grade fever and an elevated serum CRP level. These

**Figure 1** Time course of clinical manifestations of pulmonary toxicity. The leucocyte counts are expressed as the range in the six cases. The arrowheads indicate the starting day of each CHOP therapy

**Figure 2** Interstitial lung shadow revealed by (**A**) chest radiography and (**B**) chest CT scan of case 1

© Cancer Research Campaign 1998 

findings had not been observed during the prior CHOP courses. Seven and a half days (median) after the start of the next CHOP course, all six cases developed a low-grade fever with an elevated serum CRP level. At that time, only one of the six cases had neutropenia ($0.9 \times 10^9$ $l^{-1}$), and the median neutrophil count for the six cases was $7.2 \times 10^9$ $l^{-1}$. During the subsequent clinical course, only two cases (including the above neutropenic case) had neutropenia ($\leq 1 \times 10^9$ $l^{-1}$), whose duration was only 2 days in both cases. In all cases, a high-grade fever and hypoxaemia with an interstitial lung shadow on the CT scan and/or chest radiography developed 5 and 9 days (median) after the first day of this low-grade fever respectively. CT scanning was better able to detect the interstitial lung shadow in most cases. A non-productive cough and fine crackles on chest examination developed in four and two cases, respectively, usually after the hypoxaemia had developed. Eosinophilia was not observed in any cases. The radiological films of a representative case are shown in Figure 2.

All cases were initially treated with antibiotics, with or without an antifungal agent empirically. However, because this therapy was ineffective, and micro-organisms were not detected in repeated examinations, it was replaced with corticosteroid therapy. Five cases were treated with 1 g of methylprednisolone daily for 3 consecutive days, followed by prednisolone (PSL) (1 mg $kg^{-1}$) daily, which was reduced according to the clinical response. The remaining one case was treated with PSL (1 mg $kg^{-1}$) daily from the beginning. The pulmonary disease of all six cases was resolved by the corticosteroid therapy. Lymphocyte transformation tests using CPA, DOX, VCR and G-CSF were performed in five of the six cases. The results were all negative except for a weakly positive response to CPA in case 5. After the lung toxicity had been resolved, two of the six cases (cases 2 and 3) were readministered G-CSF with a different chemotherapeutic regimen, in which the dose and duration of G-CSF were minimized (the leucocyte count did not exceed $5 \times 10^9$ $l^{-1}$). The pulmonary toxicity did not recur.

We then compared the incidence of pulmonary toxicity between the present G-CSF combined CHOP therapy and the standard CHOP therapy consisting of the same chemotherapeutic drugs and doses given every 3 weeks without G-CSF. Our literature review found almost no reports of symptomatic pulmonary toxicity in NHL patients treated with the CHOP therapy (McKelvey et al, 1976; Jones et al, 1983; Shapiro et al, 1991; Fisher et al, 1993). The exception was a study that reported that 5 out of 174 patients (3%) developed mild pulmonary toxicity (ECOG grade 1 or 2) and 2 out of 174 patients (1%) showed severe lung toxicity (Gordon et al, 1992). Even compared with the results of this report, our present G-CSF combined CHOP therapy showed a significantly higher incidence of pulmonary toxicity (subjects with any toxicity grade, 7 out of 174 vs 6 out of 52, $P = 0.04$; subjects with severe toxicity grade (ECOG grades 3–5), 2 out of 174 vs 5 out of 52, $P = 0.002$). We also note that none of the 49 NHL patients we treated with CHOP therapy without G-CSF, between 1985 and 1991, developed pulmonary toxicity.

**Table 2** Comparison of characteristics between subjects who developed lung toxicity and subjects who did not

| | Lung toxicity | |
|---|---|---|
| | **Positive (*n* = 6)** | **Negative (*n* = 46)** |
| Pretreatment characteristics | | |
| Age (years) | 53.2 ± 15.8 | 52.8 ± 15.3 |
| Gender (M/F) | 4/2 | 22/24 |
| NHL[a] | | |
| Grade (H/I/L/M) | 1/3/1/1 | 1/34/8/3 |
| Phenotype (B/T) | 5/1 | 36/10 |
| Stage (I/II/III/IV) | 0/0/4/2 | 2/7/17/20 |
| Serum level | | |
| LDH (IU $l^{-1}$) | 856 ± 790 | 725 ± 831 |
| sIL-2R (U $ml^{-1}$) | 2870 ± 1449 | 3212 ± 2035 |
| CRP (mg 100 $ml^{-1}$) | 4.1 ± 3.8 | 4.6 ± 6.0 |
| Therapy[b] | | |
| Cycle | 4.0 ± 1.4 | 5.3 ± 2.1 |
| Interval (days) | 14.6 ± 0.7 | 15.0 ± 3.2 |
| Drugs | | |
| CPA (mg $m^{-2}$) | 720 ± 103 | 700 ± 115 |
| DOX (mg $m^{-2}$) | 48 ± 7 | 47 ± 7 |
| VCR (mg $m^{-2}$) | 1.2 ± 0.3 | 1.2 ± 0.2 |
| PSL (mg $m^{-2}$) | 255 ± 12 | 236 ± 27 |
| G-CSF (μg $kg^{-1}$ | 22.8 ± 6.9 | 19.3 ± 5.5 |
| Leucocyte count[c] | | |
| Nadir value ($\times 10^9$ $l^{-1}$) | 2.9 ± 1.4 | 3.2 ± 2.0 |
| Peak value ($\times 10^9$ $l^{-1}$) | 29.5 ± 5.0[d] | 23.2 ± 8.5 |
| Occurrence of lung toxicity as a function of peak value | | |
| Peak value ≥ 23.0 × $10^9$ $l^{-1}$ | 6[e] | 23 |
| Peak value < 23.0 × $10^9$ $l^{-1}$ | 0 | 23 |

Data are expressed as mean ± s.d. or case number. [a]Histological grade by the Working Formulation (H, high grade; I, intermediate grade; L, low grade; M, miscellaneous), immunophenotype (B, B-cell type; T, T-cell type) and clinical stage. [b]Administered therapy cycle, average interval between each cycle and dose of drugs per therapy cycle. [c]Definitions of the nadir and peak values are described in Patients and methods. [d]$P = 0.048$ (Mann–Whitney *U*-test). [e]$P = 0.020$ ($\chi^2$ test). LDH, lactate dehydrogenase.

### Comparison of characteristics between subjects who did/did not develop pulmonary toxicity (Table 2)

In the 52 patients who received the G-CSF-combined CHOP therapy, there were no significant differences in the pretreatment characteristics between the subjects who developed pulmonary toxicity and those who did not. Although the pulmonary toxicity developed only in subjects with advanced NHL (stage III or IV), this was not statistically significant ($P > 0.2$). The subjects who developed pulmonary toxicity received a slightly smaller number of therapy cycles compared with the other subjects ($P > 0.1$). The doses of chemotherapeutic drugs and G-CSF per therapy cycle and the therapy interval did not differ between the two groups. The only difference was the peak leucocyte count. The subjects who developed pulmonary toxicity had a significantly higher peak leucocyte count than the subjects who did not ($P = 0.048$). Further, the pulmonary toxicity developed only in subjects who had a peak leucocyte count above $23.0 \times 10^9$ $l^{-1}$ ($\geq 23.0 \times 10^9$ $l^{-1}$, 6 out of 29 subjects (21%); $< 23.0 \times 10^9$ $l^{-1}$, 0 out of 23 subjects). This difference was statistically significant ($P = 0.020$). Exactly the same results were obtained when the peak neutrophil count was used for the analysis instead of the peak leucocyte count ($P = 0.020$ at a cut-off peak neutrophil count of $20.0 \times 10^9$ $l^{-1}$).

 © Cancer Research Campaign 1998

### DISCUSSION

The diagnosis of drug-induced pulmonary toxicity in the present cases is supported by the following findings: significant neutropenia was not observed when the pulmonary symptoms developed; repeated tests for micro-organisms were negative; there was probably no pulmonary invasion by the NHL (none of the six cases had pulmonary invasion by NHL before the CHOP therapy, and interstitial lung disease developed when the NHL responded well to the CHOP therapy); interstitial lung disease developed with the same time course; and all cases were successfully treated with corticosteroids. In almost all of the previously reported cases who developed pulmonary toxicity after receiving chemotherapy and G-CSF, the applied chemotherapy alone can cause pulmonary toxicity at a considerable incidence (Iki et al, 1993; Katoh et al, 1993; Matthews, 1993; Okubo et al, 1993; Dirix et al, 1994; Lei et al, 1994; Niitsu et al, 1995). Therefore, the contribution of G-CSF to the pulmonary toxicity in these prior cases is difficult to define clearly. Meanwhile, our literature review indicated that the standard CHOP therapy, given every 3 weeks without G-CSF, does not cause pulmonary toxicity. Or, if it does, the induced toxicity is usually mild and observed in a small percentage of patients. The same conclusion was reached by a review by other authors (Shapiro et al, 1991). It is also noted that none of our NHL patients treated with the standard CHOP therapy without G-CSF developed pulmonary toxicity. In contrast, the present G-CSF-combined CHOP therapy given every 2 weeks showed a significantly higher incidence of pulmonary toxicity compared with the standard CHOP therapy.

The above findings indicate that G-CSF and/or the increased dose intensity of the chemotherapy, which was enabled by the G-CSF usage, were associated with the development of pulmonary toxicity in our cases. Further, of the total 52 patients who received the G-CSF-combined CHOP therapy, the dose and interval of CHOP therapy did not differ between the subjects who developed pulmonary toxicity and those who did not. Also, the number of CHOP therapy cycles was slightly smaller in the former group. On the other hand, a high peak leucocyte count showed a statistically significant association with the development of pulmonary toxicity; the toxicity developed only in subjects who had a peak leucocyte count above $23.0 \times 10^9\ l^{-1}$ (6 out of 29 subjects, 21%). These findings suggest that the effect of G-CSF, not the CHOP dose intensity, was the main determinant of the development of pulmonary toxicity in the present cases. These data also allow speculation that, in addition to differences in the chemotherapy (kinds of drugs and number of cycles), the difference in the degree of G-CSF-induced leucocytosis may explain why G-CSF-associated pulmonary toxicity was not apparent in earlier randomized studies (Bastion et al, 1994*a*).

Based on the present data, we speculate two possible mechanisms for the development of pulmonary toxicity in G-CSF-combined chemotherapy. First, an increased number of functionally activated neutrophils may play a role (such as by releasing reactive oxygen) in the development of lung damage that probably has already been initiated subclinically by the administered chemotherapeutic drugs. We performed BAL in four out of six cases who developed pulmonary toxicity, and lymphocytes, not neutrophils, were the predominant cells in the BAL fluid in all cases (data not shown). This finding may conflict with this mechanism. However, because the BAL fluid does not necessarily reflect the interstitial cell components (Lugano et al, 1982; Paradis et al, 1986), there is a possibility that neutrophils accumulated in the interstitial tissue and caused the pulmonary damage. The second mechanism is based on the concept that the subjects who had a higher peak leucocyte count during the therapy may merely be an indication that the biological effect of G-CSF was stronger in these subjects compared with the other subjects. Besides granulopoiesis, various actions of G-CSF that are not mediated by neutrophils have been reported, some of which may contribute to the pathogenesis of lung diseases and tissue fibrosis (Vaillant et al, 1993; Pei et al, 1996). Therefore, G-CSF may exacerbate the lung damage caused by chemotherapy without mediation by neutrophils in subjects with a high peak leucocyte count (i.e. due to a strong biological effect of G-CSF). As shown in Figure 1, when the symptoms of pulmonary toxicity developed, the peak leucocyte count had passed, but G-CSF continued to be administered. This finding may support the second mechanism proposed above. However, the first mechanism is still possible because PSL in the CHOP therapy (given on days 1–5) may simply delay the development of clinically apparent pulmonary toxicity, which appeared 7.5 days (median) after starting the CHOP therapy. An idiosyncratic reaction to G-CSF is a very unlikely mechanism for the pulmonary toxicity in the present cases. This is because the toxicity was associated with a high leucocyte count, the toxicity did not recur in two cases who were readministered G-CSF, and the lymphocyte transformation response to G-CSF was negative.

The pulmonary toxicity that develops after G-CSF-combined chemotherapy may be fatal (Iki et al, 1993; Katoh et al, 1993). Therefore, prevention, early detection and proper treatment of such cases is extremely important. Assuming that an idiosyncratic mechanism is very unlikely, dose modification of G-CSF seems useful for preventing this toxicity. Our data on the peak leucocyte count indicate that the G-CSF dose should be limited to assure that the leucocyte count does not exceed $23.0 \times 10^9\ l^{-1}$ in the G-CSF-combined CHOP therapy. In other protocols using different combinations and doses of chemotherapeutic drugs, the optimal limit for the G-CSF dose to prevent pulmonary toxicity may differ. To verify this point, various data, including the peak leucocyte count with each cycle of therapy and the G-CSF dose, should be compared between the subjects who did and did not develop toxicity on other protocols. To date, detailed data of such analyses are not available in the literature. We believe that the time course of the clinical manifestations shown in Figure 1 will aid in the diagnosis of pulmonary toxicity associated with G-CSF-combined chemotherapy. Further, our data suggest that, if G-CSF-associated pulmonary toxicity develops, early application of high-dose corticosteroid deserves to be considered in addition to discontinuation of G-CSF.

### REFERENCES

Bastion Y, Reyes F, Bosly A, Gisselbrecht C, Yver A, Gilles E, Maral J and Coiffier B (1994*a*) Possible toxicity with the association of G-CSF and bleomycin. *Lancet* **343**: 1221–1222

Bastion Y and Coiffier B (1994*b*) Pulmonary toxicity of bleomycin: is G-CSF a risk factor? *Lancet* **344**: 474

Dirix LY, Schrijvers D, Druwe P, Van Den Brande J, Verhoeven D and Van Oosterom AT (1994) Pulmonary toxicity and bleomycin. *Lancet* **344**: 56

Fisher RI, Gaynor ER, Dahlberg S, Oken MM, Grogan TM, Mize EM, Glick JH, Coltman CJ and Miller TP (1993) Comparison of a standard regimen (CHOP) with three intensive chemotherapy regimens for advanced non-Hodgkin's lymphoma. *N Engl J Med* **328**: 1002–1006

© Cancer Research Campaign 1998 

Gordon LI, Harrington D, Andersen J, Colgan J, Glick J, Neiman R, Mann R, Resnick GD, Barcos M, Gottlieb A and O'Connell M (1992) Comparison of a second-generation combination chemotherapeutic regimen (m-BACOD) with a standard regimen (CHOP) for advanced diffuse non-Hodgkin's lymphoma. *N Engl J Med* **327**: 1342–1349

Iki S, Yoshinaga K, Ohbayashi Y and Urabe A (1993) Cytotoxic drug-induced pneumonia and possible augmentation by G-CSF-clinical attention. *Ann Hematol* **66**: 217–218

Jones SE, Grozea PN, Metz EN, Haut A, Stephens RL, Morrison FS, Talley R, Butler JJ, Byrne GJ, Hartsock R, Dixon D and Salmon SE (1983) Improved complete remission rates and survival for patients with large cell lymphoma treated with chemoimmunotherapy. A Southwest Oncology Group Study. *Cancer* **51**: 1083–1090

Katoh M, Shikoshi K, Takada M, Umeda M, Tsukahara T, Kitagawa S and Shirai T (1993) Development of interstitial pneumonitis during treatment with granulocyte colony-stimulating factor. *Ann Hematol* **67**: 201–202

Kreisman H and Wolkove N (1992) Pulmonary toxicity of antineoplastic therapy. *Semin Oncol* **19**: 508–520

Lei KI, Leung WT and Johnson PJ (1994) Serious pulmonary complications in patients receiving recombinant granulocyte colony-stimulating factor during BACOP chemotherapy for aggressive non-Hodgkin's lymphoma. *Br J Cancer* **70**: 1009–1013

Lugano EM, Dauber JH and Daniele RP (1982) Acute experimental silicosis. Lung morphology, histology, and macrophage chemotaxin secretion. *Am J Pathol* **109**: 27–36

Matthews JH (1993) Pulmonary toxicity of ABVD chemotherapy and G-CSF in Hodgkin's disease: possible synergy. *Lancet* **342**: 988

McKelvey EM, Gottlieb JA, Wilson HE, Haut A, Talley RW, Stephens R, Lane M, Gamble JF, Jones SE, Grozea PN, Gutterman J, Coltman C and Moon TE (1976) Hydroxyldaunomycin (Adriamycin) combination chemotherapy in malignant lymphoma. *Cancer* **38**: 1484–1493

Niitsu N and Umeda M (1995) COP-BLAM regimen combined with granulocyte colony-stimulating factor and high-grade non-Hodgkin's lymphoma. *Eur J Haematol* **55**: 88–92

Ohsaka A, Kitagawa S, Sakamoto S, Miura Y, Takanashi N, Takaku F and Saito M (1989) In vivo activation of human neutrophil functions by administration of recombinant human granulocyte colony-stimulating factor in patients with malignant lymphoma. *Blood* **74**: 2743–2748

Oken MM, Creech RH, Tormey DC, Horton J, Davis TE, McFadden ET and Carbone PP (1982) Toxicity and response criteria of the Eastern Cooperative Oncology Group. *Am J Clin Oncol* **5**: 649–655

Okubo Y and Nakazawa K (1993) Recombinant G-CSF and the interstitial pneumonia during MACOP-B therapy in two cases of non-Hodgkin's lymphoma. *Rinsho Ketsueki* **34**: 473–477

Paradis IL, Dauber JH and Rabin BS (1986) Lymphocyte phenotypes in bronchoalveolar lavage and lung tissue in sarcoidosis and idiopathic pulmonary fibrosis. *Am Rev Respir Dis* **133**: 855–860

Pei XH, Nakanishi Y, Takayama K, Yatsunami J, Bai F, Kawasaki M, Wakamatsu K, Tsuruta N, Mizuno K and Hara N (1996) Granulocyte-colony stimulating factor promotes invasion by human lung cancer cell lines in vitro. *Clin Exp Metastasis* **14**: 351–357

Shapiro CL, Yeap BY, Godleski J, Jochelson MS, Shipp MA, Skarin AT and Canellos GP (1991) Drug-related pulmonary toxicity in non-Hodgkin's lymphoma. Comparative results with three different treatment regimens. *Cancer* **68**: 699–705

Vaillant P, Muller V, Martinet Y and Martinet N (1993) Human granulocyte- and granulocyte–macrophage-colony stimulating factors are chemotactic and 'competence' growth factors for human mesenchymal cells. *Biochim Biophys Res Commun* **192**: 879–885

Welte K, Gabrilove J, Bronchud MH, Platzer E and Morstyn G (1996) Filgrastim (r-metHuG-CSF): the first 10 years. *Blood* **88**: 1907–1929

*© Cancer Research Campaign 1998*